# Exercise intolerance and systemic manifestations of pulmonary emphysema in a mouse model

**DOI:** 10.1186/1465-9921-10-7

**Published:** 2009-01-28

**Authors:** Lars Lüthje, Tobias Raupach, Hellmuth Michels, Bernhard Unsöld, Gerd Hasenfuss, Harald Kögler, Stefan Andreas

**Affiliations:** 1 Kardiologie und Pneumologie, Georg-August-Universität, Göttingen, Germany; 2 Lungenfachklinik Immenhausen, Kassel, Germany

## Abstract

**Background:**

Systemic effects of chronic obstructive pulmonary disease (COPD) significantly contribute to severity and mortality of the disease. We aimed to develop a COPD/emphysema model exhibiting systemic manifestations of the disease.

**Methods:**

Female NMRI mice were treated 5 times intratracheally with porcine pancreatic elastase (emphysema) or phosphate-buffered saline (control). Emphysema severity was quantified histologically by mean linear intercept, exercise tolerance by treadmill running distance, diaphragm dysfunction using isolated muscle strips, pulmonary hypertension by measuring right ventricular pressure, and neurohumoral activation by determining urinary norepinephrine concentration.

**Results:**

Mean linear intercept was higher in emphysema (260.7 ± 26.8 μm) than in control lungs (24.7 ± 1.7 μm). Emphysema mice lost body weight, controls gained weight. Running distance was shorter in emphysema than in controls. Diaphragm muscle length was shorter in controls compared to emphysema. Fatigue tests of muscle strips revealed impaired relaxation in emphysema diaphragms. Maximum right ventricular pressure and norepinephrine were elevated in emphysema compared to controls. Linear correlations were observed between running distance changes and intercept, right ventricular weight, norepinephrine, and diaphragm length.

**Conclusion:**

The elastase mouse model exhibited severe emphysema with consecutive exercise limitation, and neurohumoral activation. The model may deepen our understanding of systemic aspects of COPD.

## Background

Chronic obstructive pulmonary disease (COPD) is a major cause of death and disability worldwide [1]. The treatment of COPD improves lung function, yet it is unlikely to slow the steady downhill course of the disease or even reduce mortality [2]. Notably exercise intolerance is a central symptom in COPD patients and closely relates to quality of life and prognosis [3-5].

Although COPD affects primarily the lungs it recently became clear that systemic effects of COPD significantly contribute to its severity and mortality [1,6]. These systemic effects relate to tobacco smoke, chronic inflammation and oxidative stress [1,7,8], peripheral as well as diaphragm muscle dysfunction [9], pulmonary hypertension [10], the renin-angiotensin system [11] and the autonomic nervous system [12,13].

Although no single animal model recapitulates human COPD in its entirety, animal models are fundamental to COPD research [14]. Instillation of elastase into animal lungs has been a reliable means of quickly generating airspace enlargement [15]. The elastase-induced emphysema mouse model is well established [15-18] and with the advent of genetic engineering offers variable opportunities to dissect pathogenetic pathways [19].

A number of studies, mainly in emphysematous hamsters, have investigated the effect of exercise on ventilatory muscle, pulmonary function and pulmonary hypertension [18,20-22]. However, exercise capacity or activity has not been evaluated in these studies. One study has shown that emphysema causes skeletal muscle dysfunction but no effect on activity was noted [23]. Thus, to our knowledge, exercise intolerance as well as sympathetic activation has never been demonstrated in an emphysema model.

We aimed to develop a murine emphysema model exhibiting systemic manifestations of COPD. We hypothesized that severe pulmonary emphysema results in exercise intolerance and neurohumoral activation. Furthermore we aimed to investigate as additional parameters weight loss, diaphragm dysfunction, and pulmonary hypertension.

## Methods

### Study protocol

The investigations complied with the National Research Council guide for the care and use of laboratory animals (National Academy Press, Washington, D.C. 1996). The initial study protocol comprised of a single elastase administration as detailed below (protocol 1). This protocol was extended in protocol 2 to a total of 5 elastase administrations with 1 week as recovery period between individual instillations. Each protocol started with 12 mice for elastase treatment and 8 control mice. In a second series of protocol 2 another 12 elastase and 8 control mice were treated to gain additional information on diaphragm function and right ventricular mass and haemodynamics 6 months after the last elastase treatment.

### Experimental animals

Female NMRI mice were used (body weight 20–25 g, Harlan-Winkelmann, Borchen, Germany). NMRI mice represent a robust outbred mouse strain that possesses higher endogenous anti-protease activity as, e.g., mice from the C57 background [24]. Thus, we used NMRI mice, assuming they would exhibit better tolerance toward the acute toxic effects of intratracheal elastase administration. Female mice were chosen, because they usually gain less weight than males during prolonged observation periods. Human COPD occurs more frequently in men than women. This, however, does not reflect intrinsic biological differences but is generally accepted to be a manifestation of differing smoking behaviour.

### Emphysema induction

For elastase instillation mice were anaesthetized with isoflurane, intubated orotracheally and ventilated via ventilator/respirator (Mini-Vent Small Animal Ventilators, Harvard Apparatus/Hugo Sachs Elektronik, March-Hugstätten, Germany). Experimental animals received 5 U/100 g body weight (protocol 1) and 3.3 U/100 g body weight (protocol 2) porcine pancreatic elastase (Sigma-Aldrich, Taufkirchen, Germany) dissolved in 50 μl phosphate-buffered saline solution (PBS). Control animals received 50 μl of PBS. The respective solution was administered intratracheally via a tube followed by 200 μl of air for an even distribution of the liquid throughout both lungs. Afterwards mice were kept on a warm plate (30°C) until restoration of spontaneous breathing and extubated.

### Body weight, norepinephrine concentration, and exercise tolerance

Mice were maintained on a 12/12-hour light-dark cycle and were fed ad libitum. The body weight was determined daily throughout the study period till to the final experiments. At baseline as well as 7 days after the single (protocol 1) or last (protocol 2) intervention, each mouse was placed in a metabolic cage (Tecniplast GmbH, Wilhelmshaven, Germany) for 24 hours, provided with food and water ad libitum. Urine was collected, acidified and stored at -70°C until determination of norepinephrine concentration (NE). NE was measured by high-performance liquid chromatography and normalised to urinary creatinine.

For determination of the exercise tolerance treadmill exercise tests were performed using a motorized rodent treadmill equipped with a shock-plate incentive (Exer-6M Open Treadmill, Columbus Instruments, Columbus, Ohio, USA). To habituate the mice to the treadmill three exercise tests were performed with one day of rest between the tests within one week before starting the experiments. The final exercise test was used as baseline test and the distance covered calculated as the time-speed-integral up to the point of exhaustion. Six days after each (protocol 1 and 2) intubation procedure further exercise tests for comparison with baseline were performed. Treadmill speed (start value: 10 m/min) was increased by 1 m/min every two minutes with no upper limit. The slope of the treadmill was kept constant at 5°. Criteria for the end of the test were exhaustion of the animals defined as inability to return to the treadmill belt despite repetitive electric stimulation.

### Organ preparation and assessment of functional lung parameters

During the third week after the (final) intratracheal treatment, organ preparation and measurement of lung function parameters for the calculation of static compliance were performed. Mice were anaesthetized deeply and their thorax opened. Blood was collected by cardiac puncture to achieve a largely bloodless lung. A tracheal incision was performed facilitating the insertion and fixation of a blunted cannula. The trachea and both lungs were mobilized and removed from the thorax in a whole. For stable experimental conditions the lungs were submerged in a water bath. The cannula was connected with an infusion pump and the lungs were inflated twice at a rate of 15 ml/h to a maximum airway pressure of 25 cm H_2_O measured with an interposed pressure transducer (Linearcorder Mark 7, Harvard Apparatus/Hugo-Sachs-Elektronik, March-Hugstetten, Germany). Then the tubing system was opened to the atmosphere, allowing air to leak via a cannula out of the system. The corresponding volume-pressure curves were digitally recorded and served as basis for the calculation of the static compliance. Afterwards, the right lung was kept in 10% formalin fixation liquid at a constant pressure of 25 cm H_2_O for at least 24 hours, followed by histological assessment.

### Histological analysis

The fixed lungs were embedded in paraffin and sections of five micrometers were cut. Tissue sections were stained with haematoxylin and eosin. For morphometric analysis a computerized microscope with a high-resolution video camera (BX S1, Olympus, Tokyo, Japan, magnification 10×) was used. For mean linear intercept (Lm) determination 10 sagittal sections spanning the entire lung and containing apical as well as basal areas of the organ were randomly selected from each mouse. The Lm as indicator of air space size was calculated from counting lines of defined length as previously described [25]. Briefly the lines were randomly placed on every of the 10 lung sections and the number of intercepts crossing the lines counted. The mean linear intercept is calculated from the length of the lines multiplied by the number of the lines divided by the sum of all counted intercepts.

### Determination of right ventricular haemodynamics and mass

*In vivo *haemodynamic analysis was performed using a 1.4 F microtip catheter (model SPR-839, Millar Instruments Inc., Houston, USA) placed through the right ventricular (RV) apex in the open-chest, isoflurane-anesthetized mouse and positioned along the longitudinal axis of the RV to record chamber pressure by micromanometry. Afterwards mice were euthanized with isoflurane and lungs and hearts were rapidly excised. Right and left ventricular weight were measured and normalized with regard to tibia length. This has proven superior to normalization with regard to body weight in animals of differing weights [26].

### Diaphragm muscle function

Following excision of hearts and lungs as described above the empty chest was rinsed with ice-cold modified Krebs-Henseleit (K-H) buffer solution containing (in mM): Na^+ ^140.5, K^+ ^5.1, Mg^2+ ^1.2, Ca^2+ ^0.25, Cl^- ^124.9, SO_4_^2- ^1.2, PO_4_^3- ^2.0, HCO_3_^- ^20, glucose 10, butanedionemonoxime (BDM) 20, pH 7.4. A ring of the chest wall with the diaphragm attached was excised and cleared from blood in ice-cold K-H buffer solution. Longitudinal muscle strips of 0.8 – 1 mm width were dissected from the left posterior quadrant of the diaphragm such that on the one end a piece of the adjacent rib and on the other end a piece of the tendinous center of the diaphragm were left. Preparations were mounted isometrically between a wire loop connected to a force transducer (Scientific Instruments, Heidelberg, Germany) and a hook connected to a micrometer drive for length adjustment and superfused at 37°C with K-H solution equilibrated with 95% O_2_/5% CO_2_. The solution was replaced with BDM-free K-H solution containing Ca^2+ ^at 1.25 mmol/L, and electrical field stimulation was initiated at 1 Hz using biphasic pulses of 4 ms duration and 5 V amplitude (Stimulator STIM1, Scientific Instruments, Heidelberg, Germany). Preparations were stretched to a passive tension of 1 mN/mm^2 ^and allowed to functionally stabilize for 30 min. 3-s trains of electrical stimuli in 10-Hz increments (covering the range 10 – 120 Hz) were applied once per minute and the force response was recorded on a computer using LabView (National Instruments). After another 30-min period for recovery, a fatigue test was initiated consisting of 100 repetitive trains of stimuli at 100 Hz, 250 ms duration, 1/s, during which tetanic force and its decline due to fatigue were recorded. All force values were normalized to the cross-sectional area of the respective muscle preparations (mN/mm^2^) [27].

### Statistical analysis

All variables were tested for normal distribution and are given as mean ± SEM. To compensate for group differences at baseline, body weight and exercise performance were expressed as percentage of the respective baseline value. For the in-group comparison of the relative baseline value versus the relative value after the final intubation procedure a paired t-test was performed. For comparison between control and the respective emphysema groups an unpaired t-test was performed. For comparison of control and the two emphysema groups treated by different protocols a one-way ANOVA with Bonferroni's correction was applied. For the combined group (emphysema and control) and the distinct groups the change in running distance was correlated to various parameters using the Pearson coefficient. Two-tailed tests were used and significance was accepted at a value of p < 0.05.

## Results

### Single administration

During protocol 1 a total of six mice treated with elastase died. This was caused twice by pneumothorax and in four cases by haemorrhage evidenced by macroscopic evaluation. Two control mice died during the intubation procedure. Thus, a total of six mice in each group were available for analysis.

### Repetitive administration

During the first series of protocol 2 five elastase treated mice and one control mouse died. Haemorrhage caused death in one mouse and pneumothorax in another in the elastase group. A total of three mice in the elastase group and one more in the control group died during the intubation procedure. Thus, finally seven mice in each group were available for analysis. In the second series of protocol 2 three mice in the elastase group (pneumothorax in two mice and haemorrhage in one mouse) and one mouse in the control group (during intubation procedure) died. Thus, evaluation of diaphragm and RV was conducted from nine elastase treated mice and seven control mice.

### Histology

**Figure 1:**
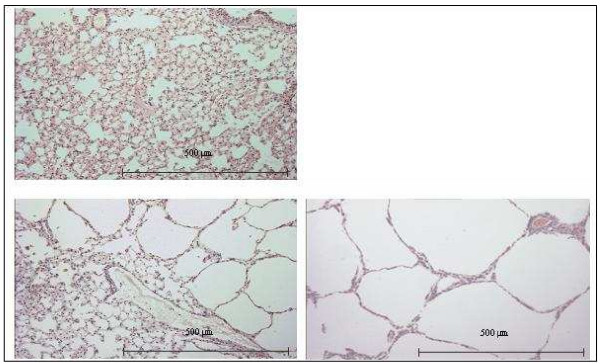
**Representative photomicrographs showing morphology of lungs treated with phosphate buffered salt (normal lung morphology; upper panel), single elastase application (lower panel on the left), and repetitive elastase instillations over a period of 5 weeks (pronounced emphysematous changes; lower panel on the right)**.

The mean linear intercept was significantly higher by single elastase administration compared to control (108.0 ± 14.7 μm vs. 30.2 ± 0.8 μm; p < 0.05). Repetitive elastase instillation resulted in a more pronounced emphysema, reflected by a significantly higher mean linear intercept (260.7 ± 26.8 μm) as compared to the single treatment regimen (p < 0.0001; table 1, for example see figure 1).

**Table 1:** Lung, heart and diaphragm parameters

	**elastase**	**controls**	**P value**
**Mean linear intercept [μm]**	260.7 ± 26.8	24.7 ± 1.7	<0.001
**static compliance [μl/cm H_**2**_O]**	185.1 ± 17.1	48.5 ± 5.0	<0.001
**Diaphragm muscle length [mm]**	5.1 ± 0.1	6.8 ± 0.3	<0.001
**Tibia length [mm]**	19.6 ± 0.2	19.9 ± 0.1	0.2
**Heart weight [mg]**	145.4 ± 5.4	148.7 ± 6.4	0.7
**Right ventricular weight [mg]**	44.0 ± 3.2	34.3 ± 3.2	0.055
**Heart weight/tibia length [mg/mm]**	7.4 ± 0.3	7.5 ± 0.3	0.95
**RV weight/tibia length [mg/mm]**	2.2 ± 0.2	1.7 ± 0.2	<0.05
**dp/dt max [mmHg/s]**	2389 ± 102	1143 ± 159	<0.0001
**RV peak systolic pressure [mmHg]**	38.8 ± 1.3	21.8 ± 1.2	<0.0001
**RV end-diastolic pressure [mmHg]**	4.6 ± 0.5	3.1 ± 0.3	<0.05

Lung parameters derived from series 1 of protocol 2; diaphragm muscle length, tibia length, heart weight and hemodynamics derived from series 2 of protocol 2

### Body weight

The single administration protocol had no significant effect on the body weight change neither of the elastase-treated nor of the control mice. After the repetitive administration protocol, however, elastase-treated mice lost body weight compared to baseline (94.9 ± 2.4%), whereas control mice gained weight (110.4 ± 2.8%), resulting in a significant difference between emphysema and control animals (p = 0.001).

### Norepinephrine concentration

**Figure 2:**
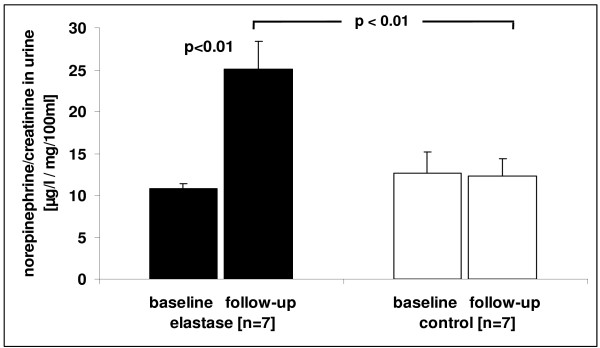
**Norepinephrine (NE) concentration in urine of elastase treated mice (protocol 2) and controls**. Repetitive administration of elastase lead to a significantly higher norepinephrine concentration at follow-up compared to control animals.

The single administration protocol did not result in a significant difference of urinary norepinephrine concentration between the elastase and control group (18.3 ± 1.3 μg/l/mg/100 ml creatinine vs. 16.9 ± 0.8 μg/l/mg/100 ml creatinine; p = 0.36). In contrast, the repetitive administration protocol lead to a significant difference in norepinephrine concentration at follow-up between elastase and control animals (p < 0.01; figure 2).

### Exercise tolerance

**Figure 3:**
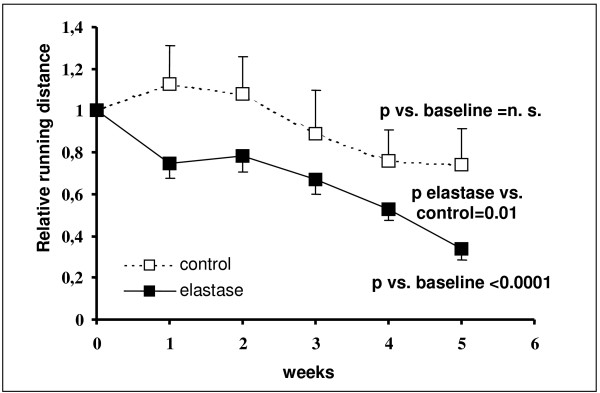
**Exercise tolerance of elastase treated (protocol 2) and control mice**. Shown are the running distances at the follow-up evaluations after the respective elastase treatment normalized to the baseline running distance (week 0). The relative running distance after the last elastase instillation was significantly shorter in the elastase group than in the control group.

No significant exercise performance change was observed between elastase and control mice after the single administration protocol. Following repetitive elastase instillation, however, the relative running distance after the last elastase instillation was significantly reduced in the elastase group (29.7 ± 8.7% of baseline; p < 0.0001) compared with the control group (79.1 ± 14.0% of baseline; p = n. s.; elastase vs. control p = 0.01; figure 3).

**Figure 4:**
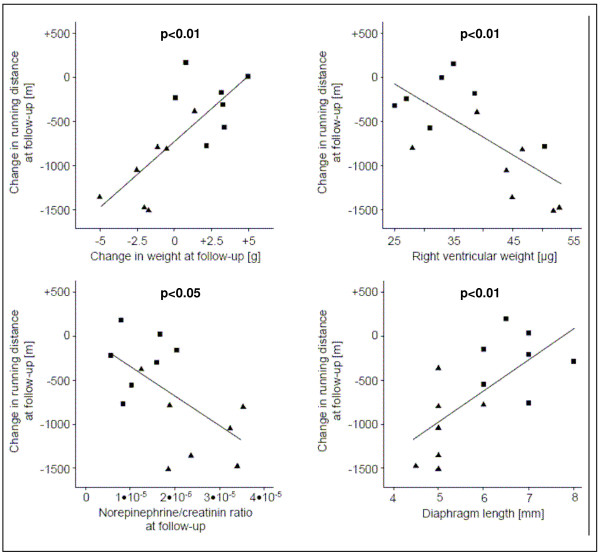
**Correlation analysis between changes in running distance and body weight change, right ventricular weight, norepinephrine concentration, and diaphragm length**.

Correlation analysis for the combined groups revealed linear relationships between changes in running distance and changes in body weight (r = 0.76; p < 0.01), mean linear intercept (r = -0.64; p < 0.05), right ventricular weight (r = -0.71; p < 0.01), diaphragm length (r = 0.69; p < 0.01), and norepinephrine concentration at follow-up (r = -0.59; p < 0.05; figure 4). Correlation analysis in the distinct groups, showed a trend for changes in running distance and changes in body weight (r = 0.74; p < 0.1), as well as right ventricular weight (r = -0.68; p < 0.1) in the elastase group, but no such trend in the control group.

### Static compliance

Single elastase instillation resulted in a non-significantly higher static compliance of 66.3 ± 3.9 μl/cm H_2_O compared to controls with 43.5 ± 5.2 μl/cm H_2_O. Compared to the single treatment regimen the static compliance was significantly higher after repetitive elastase instillation (p < 0.0001 vs. single elastase treatment; table 1).

### RV haemodynamics and mass

**Figure 5:**
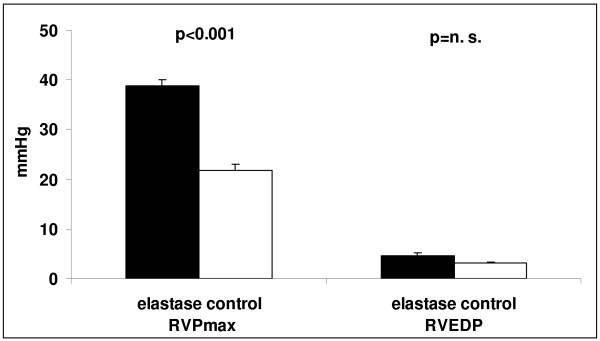
***In vivo *analysis of peak systolic RV pressure (RVP max) and RV end-diastolic pressure (RVEDP) of control and elastase treated (protocol 2) mice, indicating pulmonary hypertension of emphysematous mice**.

Evaluation of the RV parameters was only done in repetitively treated animals 6 months after the last elastase treatment. RV end-diastolic pressure was mildly, but not significantly, higher in elastase-treated compared to control mice. RV peak systolic pressure, however, was nearly doubled in emphysema compared to control mice (p < 0.0001, table 1 and figure 5), indicating pulmonary hypertension. RV mass normalized to tibia length of the animals was significantly higher in emphysema animals than in controls (p < 0.05; table 1), confirming RV pressure-induced hypertrophy.

### Diaphragm

**Figure 6:**
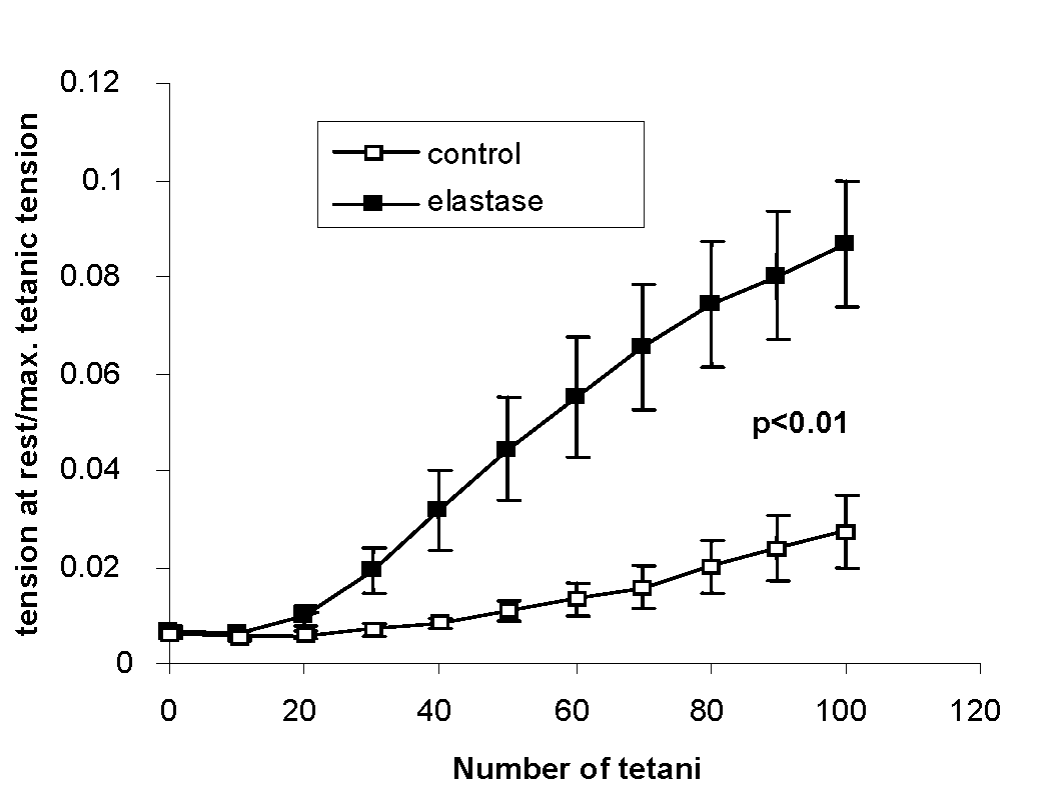
**Tension at rest normalized to maximum tetanic tension of isolated longitudinal muscle strips of control and emphysema (protocol 2) mice using repetitive trains of stimuli**. Diaphragm muscle strips of elastase treated mice showed incomplete relaxation, while relaxation was unimpaired in preparations from control mice.

Diaphragm evaluation was conducted only in repetitively treated animals 6 months after the last elastase treatment. Diaphragm muscle length was significantly shorter in elastase-treated compared to control mice (p < 0.001; table 1). The force-frequency relationship as well as the developed tension during the fatigue test of isolated diaphragm muscle strips did not differ between elastase treated mice and control mice. However, during the fatigue test diaphragm muscle strips of elastase-treated mice showed incomplete relaxation, while relaxation was unimpaired in preparations from control mice (p < 0.01; Figure 6)

## Discussion

The described model of pulmonary emphysema for the first time allows investigating exercise intolerance as a central symptom of COPD. Furthermore, our murine model shows systemic manifestations, namely reduced body weight, diaphragm dysfunction, sympathetic activation, and pulmonary hypertension that contribute to the pathophysiology and clinical appearance of COPD in man. Particularly neurohumoral activation in COPD to our knowledge has never before been observed in an animal model [13].

### Single versus repetitive elastase application

Elastase instillation was established > 40 years ago to develop a model of emphysema in hamsters [15,18]. The desired effect of elastase is limited to a narrow window of dosage, below which no significant loss of alveoli is observed, whereas a higher dose results in severe pulmonary haemorrhage [28]. Furthermore, it has been shown that elastase administration results in a heterogeneous effect on alveolar size and diameter [29]. Not surprisingly, therefore, despite clear structural and functional evidence of lung emphysema following a single administration of elastase we failed to observe general manifestations that would confirm the validity of this approach to cause a state reminiscent of human disease. Thus, we altered the study protocol to repetitive administration of reduced single doses of elastase. Repetitive intratracheal papain instillation has previously been described in rats with a high mortality (63 of 80) [30].

### Emphysema

Several animal models (including dogs, guinea pigs, monkeys, sheep, hamster, mice and rats) have been established to study COPD and emphysema [14,15,18,31]. In our study the mean linear intercept after single elastase treatment of mice was comparable to values in the literature ranging from 35 to 85 μm [17,32-35]. Furthermore the increase in compliance following elastase corroborates previous work [35]. Repetitive elastase administration, however, resulted in a significantly higher mean linear intercept and thus more severe emphysema than the single treatment regimen. The histological results were accompanied by a more severe functional change of the lung in repetitively treated mice as reflected by a significantly higher static compliance. Instillation of elastase into animal lungs is not expected to provide information on upstream events such as inflammation [36]. Yet, it was recently noticed that elastase leads to endogenous inflammation and further lung destruction over the ensuing weeks [14,37].

### Body weight

The repetitive elastase treatment protocol in this study resulted in a slight reduction of the body weight, whereas in controls body weight increased with advancing age and size of the animals. In most previous studies with single elastase application in hamsters there was no significant change in body weight [23,31,38,39]. Weight loss occurs in about 10 to 15% of patients with mild to moderate COPD and about 50% of patients with severe disease [40]. Low body weight is an important systemic manifestation of COPD and is associated with increased mortality [3]. Since we have not evaluated body composition, food intake or daily activity, we can not comment on the causes of weight loss in our mice.

### Sympathoexcitation

Recent data demonstrate that COPD patients exhibit marked neurohumoral activation [12]. The cause of this activation is not entirely clear, but the chemoreflex, baroreflex, lung inflation reflex and muscle metaboreflexes might contribute. Given the established negative connotations of neurohumoral activation in heart failure and other diseases, neurohumoral activation in COPD may well have negative consequences, namely on inflammation, cachexia, skeletal muscle dysfunction and cardiovascular disease [13]. In the present study, we found for the first time a significant increase of urinary norepinephrine concentration in emphysematous animals und thus corroborate previous findings in COPD patients [8,13]. Our model may thus serve to unravel the relationship between sympathoexcitation and COPD.

### Exercise capacity

The assessment of COPD severity based on lung function bears limitations. Therefore, additional markers for assessment of the disease have been searched for [1]. Recently the BODE index, which includes body mass index, airflow obstruction, dyspnoea and exercise capacity, has been shown to be a stronger predictor of COPD prognosis than FEV_1 _[3].

In previous emphysema animal models the effect of exercise on pulmonary function was studied. However, exercise capacity so far has not been evaluated [18,20-23,31]. In one of these studies elastase treated hamsters showed decreased oxygenation during exercise [31]. Another study found no differences in the level of activity [23]. In our study, exercise capacity was significantly lower in elastase-treated mice than in controls. As exercise capacity integrates abnormalities of pulmonary, cardiovascular and muscle function in a single measurement, the observed reduction may reflect emphysema severity, but may also originate from reduced function of other organ systems as detailed above. In our study correlation analysis reveals a relationship of borderline significance between exercise limitation and mean linear intercept, RV weight, and norepinephrine concentration. This analysis does not prove causality. However, our findings parallel clinical data and pathophysiological concepts explaining exercise limitation in COPD [1,3].

### Right ventricle

Similar to patients with COPD, our mice with severe pulmonary emphysema showed increased RV pressures as indicator for pulmonary hypertension and consecutively increased RV mass [41], indicating RV pressure-induced hypertrophy. Pulmonary hypertension has been noted previously in animal emphysema models [18,30]. Reduction of the capillary bed following pulmonary emphysema contributes to increase in pulmonary vascular pressure [10]. However, since we have not evaluated oxygen saturation we cannot comment whether hypoxemia contributes to the increase in pulmonary pressure.

### Diaphragm dysfunction

In patients with COPD, functional impairment of the diaphragm is present and associated with accelerated muscle protein degration [42]. The underlying etiology is poorly understood. Mechanical factors such as hyperinflation induced increased contractile activity of the diaphragm or systemic inflammation and oxidative stress might contribute [42,43].

In accordance with previous human and animal data we found a reduced diaphragm muscle length in the emphysema mice compared to controls [4,38]. The importance of diaphragm muscle length for respiratory breathing effort is highlighted by the positive effects of lung volume reduction surgery [4,38]. In our study evaluation of diaphragm function revealed impaired relaxation with preserved contractile function in the elastase-treated mice. Impaired relaxation of diaphragm muscle is generally considered an early index of respiratory muscle fatigue [44]. Especially at high breathing rates diaphragm relaxation is crucial, because the diaphragm must return to its optimal muscle length between each inspiration and because diaphragmatic perfusion depends on rapid relaxation [44].

### Limitations

No single animal model recapitulates human COPD in its entirety [15]. Mouse lung structure is not identical to humans. For example mice have few submucosal glands, much less airway branching, and they do not contain respiratory bronchioles – the initial site of centriacinar emphysema in man [14]. However, the mouse provides a good choice for an animal model because the mouse genome has been extensively studied and close similarities exist with the human genome [19].

Elastase instillation models are rather crude models inducing massive lung injury with a single or repetitive administration of the protease. This does not reflect the continuous low grade inflammatory process, which is believed to underlie smoking related emphysema [14,16,45]. However, elastase-induced emphysema models can be useful for studying therapeutic strategies following the insult [14,16].

## Conclusion

This is the first study showing systemic effects of COPD with pulmonary emphysema such as sympathoexcitation and exercise intolerance in an elastase mouse model. The model may help to deepen the not-well characterized impact of neurohumoral activation on pathogenesis and course of COPD with emphysema.

## Competing interests

The authors declare that they have no competing interests.

## Authors' contributions

LL, HK, GH and SA created conception and design of the study. LL and HK carried out the elastase instillation, lung preparation and lung functional testing. HM, LL, and HK carried out histological preparation and analysis, urine collection, measurement of body weight and exercise testing. LL, TR, HK, and SA drafted or helped to draft the manuscript. BU performed the experiments for evaluation of RV haemodynamics and mass. HK performed the experiments for evaluation of diaphragm function. All authors read and approved the final manuscript.

## References

[B1] Fabbri LM, Luppi F, Beghe B, Rabe KF (2006). Update in chronic obstructive pulmonary disease 2005. Am J Respir Crit Care Med.

[B2] Calverley P, Pauwels R, Vestbo J, Jones P, Pride N, Gulsvik A, Anderson J, Maden C (2003). Combined salmeterol and fluticasone in the treatment of chronic obstructive pulmonary disease: a randomised controlled trial. Lancet.

[B3] Celli BR, Cote CG, Marin JM, Casanova C, Montes de Oca M, Mendez RA, Pinto Plata V, Cabral HJ (2004). The body-mass index, airflow obstruction, dyspnea, and exercise capacity index in chronic obstructive pulmonary disease. N Engl J Med.

[B4] Gorman RB, McKenzie DK, Butler JE, Tolman JF, Gandevia SC (2005). Diaphragm length and neural drive after lung volume reduction surgery. Am J Respir Crit Care Med.

[B5] Pinto-Plata VM, Cote C, Cabral H, Taylor J, Celli BR (2004). The 6-min walk distance: change over time and value as a predictor of survival in severe COPD. Eur Respir J.

[B6] Fabbri LM, Luppi F, Beghe B, Rabe KF (2008). Complex chronic comorbidities of COPD. Eur Respir J.

[B7] Hogg JC, Chu F, Utokaparch S, Woods R, Elliott WM, Buzatu L, Cherniack RM, Rogers RM, Sciurba FC, Coxson HO, Pare PD (2004). The nature of small-airway obstruction in chronic obstructive pulmonary disease. N Engl J Med.

[B8] Raupach T, Schafer K, Konstantinides S, Andreas S (2006). Secondhand smoke as an acute threat for the cardiovascular system: a change in paradigm. Eur Heart J.

[B9] Reid MB (2001). COPD as a muscle disease. Am J Respir Crit Care Med.

[B10] Wright JL, Levy RD, Churg A (2005). Pulmonary hypertension in chronic obstructive pulmonary disease: current theories of pathogenesis and their implications for treatment. Thorax.

[B11] Stewart AG, Waterhouse JC, Billings CG, Baylis P, Howard P (1994). Effects of angiotensin converting enzyme inhibition on sodium excretion in patients with hypoxaemic chronic obstructive pulmonary disease. Thorax.

[B12] Raupach T, Bahr F, Herrmann P, Luethje L, Heusser K, Hasenfuss G, Bernardi L, Andreas S (2008). Slow breathing reduces sympathoexcitation in COPD. Eur Respir J.

[B13] Andreas S, Anker SD, Scanlon PD, Somers VK (2005). Neurohumoral activation as a link to systemic manifestations of chronic lung disease. Chest.

[B14] Wright JL, Cosio M, Churg A (2008). Animal models of chronic obstructive pulmonary disease. Am J Physiol Lung Cell Mol Physiol.

[B15] Mahadeva R, Shapiro SD (2002). Chronic obstructive pulmonary disease * 3: Experimental animal models of pulmonary emphysema. Thorax.

[B16] Dawkins PA, Stockley RA (2001). Animal models of chronic obstructive pulmonary disease. Thorax.

[B17] Foronjy RF, Mirochnitchenko O, Propokenko O, Lemaitre V, Jia Y, Inouye M, Okada Y, D'Armiento JM (2006). Superoxide dismutase expression attenuates cigarette smoke- or elastase-generated emphysema in mice. Am J Respir Crit Care Med.

[B18] Snider GL (1986). Experimental studies on emphysema and chronic bronchial injury. Eur J Respir Dis Suppl.

[B19] Shapiro SD (2007). Transgenic and gene-targeted mice as models for chronic obstructive pulmonary disease. Eur Respir J.

[B20] Heunks LM, Bast A, van Herwaarden CL, Haenen GR, Dekhuijzen PN (2000). Effects of emphysema and training on glutathione oxidation in the hamster diaphragm. J Appl Physiol.

[B21] Farkas GA, Roussos C (1984). Histochemical and biochemical correlates of ventilatory muscle fatigue in emphysematous hamsters. J Clin Invest.

[B22] Sahebjami H, Vassallo CL (1976). Exercise stress and enzyme-induced emphysema. J Appl Physiol.

[B23] Mattson JP, Poole DC (1998). Pulmonary emphysema decreases hamster skeletal muscle oxidative enzyme capacity. J Appl Physiol.

[B24] Gardi C, Cavarra E, Calzoni P, Marcolongo P, de Santi M, Martorana PA, Lungarella G (1994). Neutrophil lysosomal dysfunctions in mutant C57 Bl/6J mice: interstrain variations in content of lysosomal elastase, cathepsin G and their inhibitors. Biochem J.

[B25] Dunnill MS (1964). Evaluation of a Simple Method of Sampling the Lung for Quantitative Histological Analysis. Thorax.

[B26] Yin FC, Spurgeon HA, Rakusan K, Weisfeldt ML, Lakatta EG (1982). Use of tibial length to quantify cardiac hypertrophy: application in the aging rat. Am J Physiol.

[B27] Frey N, Frank D, Lippl S, Kuhn C, Kögler H, Barrientos T, Rohr C, Will R, Müller O, Weiler H, Bassel-Duby R, Katus H, Olson E (2008). Deficiency for calsarcin-2 increases exercise capacity in vivo by calcineurin/NFAT activation. J Clin Invest.

[B28] Busch RH, Lauhala KE, Loscutoff SM, McDonald KE (1984). Experimental pulmonary emphysema induced in the rat by intratracheally administered elastase: morphogenesis. Environ Res.

[B29] Ito S, Ingenito EP, Arold SP, Parameswaran H, Tgavalekos NT, Lutchen KR, Suki B (2004). Tissue heterogeneity in the mouse lung: effects of elastase treatment. J Appl Physiol.

[B30] Herget J, Palecek F, Cermakova M, Vizek M (1979). Pulmonary hypertension in rats with papain emphysema. Respiration.

[B31] Sexton WL, Poole DC (1998). Effects of emphysema on diaphragm blood flow during exercise. J Appl Physiol.

[B32] Fujita M, Ye Q, Ouchi H, Nakashima N, Hamada N, Hagimoto N, Kuwano K, Mason RJ, Nakanishi Y (2004). Retinoic acid fails to reverse emphysema in adult mouse models. Thorax.

[B33] Ishikawa T, Aoshiba K, Yokohori N, Nagai A (2006). Macrophage colony-stimulating factor aggravates rather than regenerates emphysematous lungs in mice. Respiration.

[B34] Murakami S, Nagaya N, Itoh T, Iwase T, Fujisato T, Nishioka K, Hamada K, Kangawa K, Kimura H (2005). Adrenomedullin regenerates alveoli and vasculature in elastase-induced pulmonary emphysema in mice. Am J Respir Crit Care Med.

[B35] Hantos Z, Adamicza A, Jánosi TZ, Szabari MV, Tolnai J, Suki B (2008). Lung volumes and respiratory mechanics in elastase-induced emphysema in mice. J Appl Physiol.

[B36] Shapiro SD (2000). Animal models for COPD. Chest.

[B37] Lucey EC, Keane J, Kuang PP, Snider GL, Goldstein RH (2002). Severity of elastase-induced emphysema is decreased in tumor necrosis factor-alpha and interleukin-1beta receptor-deficient mice. Lab Invest.

[B38] Marchand E, De Leyn P, Gayan-Ramirez G, Palecek F, de Bock V, Dom R, Decramer M (2000). Lung volume reduction surgery does not improve diaphragmatic contractile properties or atrophy in hamsters with elastase-induced emphysema. Am J Respir Crit Care Med.

[B39] Mattson JP, Delp MD, Poole DC (2004). Differential effects of emphysema on skeletal muscle fibre atrophy in hamsters. Eur Respir J.

[B40] Creutzberg EC, Schols AM, Bothmer-Quaedvlieg FC, Wouters EF (1998). Prevalence of an elevated resting energy expenditure in patients with chronic obstructive pulmonary disease in relation to body composition and lung function. Eur J Clin Nutr.

[B41] Weitzenblum E (2003). Chronic cor pulmonale. Heart.

[B42] Ottenheijm CA, Heunks LM, Dekhuijzen PN (2007). Diaphragm muscle fiber dysfunction in chronic obstructive pulmonary disease: toward a pathophysiological concept. Am J Respir Crit Care Med.

[B43] Man WD, Hopkinson NS, Harraf F, Nikoletou D, Polkey MI, Moxham J (2005). Abdominal muscle and quadriceps strength in chronic obstructive pulmonary disease. Thorax.

[B44] Coirault C, Chemla D, Lecarpentier Y (1999). Relaxation of diaphragm muscle. J Appl Physiol.

[B45] Morris DG, Sheppard D (2006). Pulmonary emphysema: when more is less. Physiology (Bethesda).

